# A BCAM0223 Mutant of *Burkholderia cenocepacia* Is Deficient in Hemagglutination, Serum Resistance, Adhesion to Epithelial Cells and Virulence

**DOI:** 10.1371/journal.pone.0041747

**Published:** 2012-07-25

**Authors:** Dalila Mil-Homens, Arsenio M. Fialho

**Affiliations:** 1 IBB-Institute for Biotechnology and Bioengineering, Center for Biological and Chemical Engineering, Instituto Superior Técnico, Lisbon, Portugal; 2 Department of Bioengineering, Instituto Superior Técnico, Technical University of Lisbon, Lisbon, Portugal; University of Osnabrueck, Germany

## Abstract

*Burkholderia cepacia* complex (Bcc) bacteria are a problematic group of microorganisms causing severe infections in patients with Cystic Fibrosis. In early stages of infection, Bcc bacteria must be able to adhere to and colonize the respiratory epithelium. Although this is not fully understood, this primary stage of infection is believed to be in part mediated by a specific type of adhesins, named trimeric autotransporter adhesins (TAAs). These homotrimeric proteins exist on the surface of many Gram negative pathogens and often mediate a number of critical functions, including biofilm formation, serum resistance and adherence to an invasion of host cells. We have previously identified in the genome of the epidemic clinical isolate *B. cenocepacia* J2315, a novel cluster of genes putatively encoding three TAAs (BCAM0219, BCAM0223 and BCAM0224). In this study, the genomic organization of the TAA cluster has been determined. To further address the direct role of the putative TAA BCAM0223 in *B. cenocepacia* pathogenicity, an isogenic mutant was constructed via insertional inactivation. The *BCAM0223::Tp* mutant is deficient in hemagglutination, affected in adherence to vitronectin and in biofilm formation and showed attenuated virulence in the *Galleria mellonella* model of infection. Moreover, the *BCAM0223::Tp* mutant also showed a significant reduction in its resistance to human serum as well as in adherence, but not in invasion of, cultured human bronchial epithelial cells. Altogether these results demonstrate that the BCAM0223 protein is a multifunctional virulence factor that may contribute to the pathogenicity of *B. cenocepacia.*

## Introduction

The *Burkholderia cepacia* complex (Bcc) is a group of closely related Gram-negative bacteria found in the environment. Over the last decades, Bcc bacteria have emerged as highly problematic opportunistic pathogens causing severe respiratory infections in cystic fibrosis (CF) patients [1]. Currently there are 17 species described as members of the Bcc complex. *B. cenocepacia*, *B. multivorans* and *B. dolosa* are those species that occur at a greater frequency and are particularly problematic [2], [3]. Colonized Bcc bacteria are transmitted among CF-patients and are extremely resistant to antimicrobial therapies [4]. In a subset of CF patients, lung infections with Bcc lead to declining lung function, with necrotising pneumonia and a fatal speticemia termed “cepacia syndrome” [5].

Many virulence determinants associated with Bcc-lung infection have been described. Overall, they enable bacteria to attach to the surface of host cells, evade host defences and replicate either extra- or intracellularly [6], [7]. During the early stages of interaction, Bcc bacteria must interact with surface receptors of the epithelial cells, such as proteins, glycolipid receptors and mucins [8]. Although these binding events remain poorly understood, it is believed that several bacterial surface-bound proteins named adhesins, are key determinants of adherence and/or invasion of target host cells. Up to date, only the cable pili-associated adhesin (*cbl*, 22 kDa) from *B. cenocepacia* strains and a 55 kDa protein from the piliated *B. cenocepacia* strain BC7 have been described to interact with cytokeratin 13 and TNF receptor1 (TNFR1) of epithelial cells, respectively [9], [10]. However, since both proteins were absent in many pathogenic Bcc isolates [11], others uncharacterized adhesins may participate in the bacterial adherence to host cells. Among such sets of proteins are the trimeric autotransporter adhesins (TAAs), a class of adhesins produced by many Gram-negative pathogens as key virulence factors. These extracellular proteins have been shown to possess adhesive properties, thereby enabling bacterial adherence to host cells [12], [13].

Over the last years, increased attention has been devoted to the study of TAAs, revealing that they are often multifunctional virulence factors with decisive roles at different stages of bacterial infection. In fact, besides their role in adherence to extracellular matrix and host cells, TAAs are also involved in several other biological traits of pathogenic bacteria including hemagglutination, autoaggregation, cytotoxicity, serum resistance and invasion to host cells [14], [15]. TAAs are found exclusively in Gram-negative bacteria and several of them have been characterized in detail, including among others, YadA from *Yersinia enterocolitica*, the prototype for this family of proteins [16], BadA from *Bartonella henselae* [17], NadA from *Neisseria meningitidis* [18], Hia from *Haemophilus influenza* [19], an IgD-binding protein from *Moraxella catarrhalis* [20], AipA and TaaP from *Proteus mirabilis* [21], BpaA from *Burkholderia pseudomallei* [22], SadA from *Salmonella enterica* [23] and Cha from *Haemophilus* cryptic genospecies [24].

All TAAs share a common domain architecture, where each monomer comprises an integral membrane-anchored C-terminal formed by four beta strands and a surface exposed passenger domain consisting of a neck, a stalk and an N-terminal head [25]. Contrary to the stalk and the head, the C-terminal translocator domain is highly conserved among TAAs and therefore is used as the defining element of the family [26]. Although it is poorly understood, the trimerization of TAAs is a multiple-step process, in which a C-terminal beta barrel membrane pore is formed, followed by the translocation of the passenger domain to the extracellular space, via a type V protein secretion pathway (T5SS) [27]. The passenger domains confer the functionalities of the TAAs; they contain repetitive sequence motifs and are variable in sequence and lengths [27].

We have previously conducted a computational analysis of the genome sequence of *B. cenocepacia* strain J2315 (ET12 lineage) and we identified a novel cluster of genes encoding 3 potential trimeric autotransporter adhesins (TAAs) (*BCAM0219*, *BCAM0223* and *BCAM0224*), 1 outer membrane protein (*BCAM0220*), 2 sensor histidine kinases (*BCAM0218*, *BCAM0227*) and 3 transcriptional regulators (*BCAM0221*, *BCAM0222*, and *BCAM0228*) [13], [28]. This cluster, here designated TAA cluster, is located within a region that corresponds to an extension of the *cci* island previous described [29]. In addition, we have identified *BCAM0224*, a gene encoding a TAA that plays a role in adhesion and virulence [28]. In the present study, we intend to elucidate the function of the *BCAM0223* gene that is located near *BCAM0224* and encodes another predicted TAA. For that purpose, we have constructed a BCAM0223-negative *B. cenocepacia* strain and characterized its ability to adhere to ECM proteins, to form biofilm, to resist to human serum, to hemagglutinate red blood cells and to cause disease in the *G. mellonella* model. Moreover, the role of BCAM0223 in adherence to or invasion of human CF and non-CF airway epithelial cell lines was also investigated. Taken together the findings indicate that BCAM0223, from the epidemic strain *B. cenocepacia* K56-2, is a novel multifunctional TAA that has hemagglutination activity, anti-complement activity and simultaneously is required for virulence and maximal host cell adherence.

## Materials and Methods

### Bacterial Strains and Growth Conditions

*B. cenocepacia* clinical isolate K56-2 was kindly provided by Prof. J. J. LiPuma (University of Michigan, USA). Bacteria were routinely cultured in Luria–Bertani (LB) broth (Conda, Pronadisa), at 37°C with orbital agitation 250 rpm. When appropriate, media was supplemented with 150 mg trimethoprim mg/L (for *B. cenocepacia BCAM0223::Tp* mutant). For functional studies, *B. cenocepacia* and mutant were grown in 24-well polystyrene microplates (Greiner Bio-One), for 17 h under limited oxygen supply (<5% oxygen), in LB media containing NaCl (300 mM) and H_2_O_2_ (10 mM) (initial OD_640_ 0.2) at 37°C and 60 r.p.m.

### Cell Lines and Cell Culture

Two human bronchial epithelial cell lines were used; 16HBE14o- cells, which are normal lung cells expressing a functional CF transmembrane conductance regulator, and CFBE41o- cells, which are homozygous for the delta F508 mutation corresponding to a CF airway. Both cell lines were immortalized and characterized by Dr. Gruenert and co-workers [30], [31] and kindly provided for this work. Cells were routinely maintained in fibronectin/vitrogen coated flasks in Minimum Essential Medium with Earle’s salt (MEM) (Gibco) supplemented with 10% fetal bovine serum (FBS) (Lonza), 0.292 g/L L-Glutamine (Sigma) and Penicillin/Streptomycin 100 U/mL (Gibco) in a humidified atmosphere at 37°C with 5% CO_2_.

### Total RNA Isolation

Total RNA was isolated from *B. cenocepacia* K56-2 grown under the conditions described above. Cells were harvested after 17 h of growth and treated with RNAprotect Bacteria reagent (Qiagen) following the manufacturer’s instructions. Bacterial lysis was achieved by enzymatic lysis with lysozyme and proteinase K (Qiagen). Total RNA was purified from bacterial lysate using RNeasy mini kit (Qiagen), according the manufacturer’s protocol. To avoid contamination with genomic DNA, RNA was treated with RNase-free DNA digestion kit (Qiagen) in column during the purification process, for 1 h at room temperature. After the RNA isolation a second step of DNase (Qiagen) treatment was performed (RNase-free DNA digestion kit), using 1 µL DNase for 1.5 µg of RNA to be treated, during 1 h at 37°C, followed by inactivation for 5 min at 65°C, according the manufacturer’s instructions. All steps described above were executed using RNase-free material. Total RNA concentration was estimated using a UV spectrophotometer (ND-1000 UV-Vis, NanoDrop Technologies, USA).

### Reverse Transcription PCR

Reverse transcription PCR (RT-PCR) was carried out using the OneStep RT-PCR kit (Qiagen), with primers listed in Table S1, following the manufacturer’s protocol. Briefly, 800 ng of total RNA were used and combined with the reverse transcriptase/polymerase mix. The first step of amplification was carried for 30 min at 50°C to perform the reverse transcription. The next steps allows the PCR amplification and we performed 35 cycles using annealing temperatures of 54°C (primers 219–220, 223–224, 225–227) and 58°C (primers 216–218, 220–221, 224–225). The amplified RT-PCR products were run in 0.8% agarose gel. For each PCR, the appropriate controls with water and RNA in the absence of reverse transcriptase and presence of polymerase were included to ensure that the amplifications obtained were a result of cDNA and not of contaminating genomic DNA.

### Construction of a BCAM0223 -Negative Mutant

A 1.8-kb *BCAM0223* fragment was amplified by PCR (Platinium *taq* DNA polymerase, Invitrogen) from *B. cenocepacia* K56-2, using primers 223fF1 and 223fR1 (Table S1), containing *Kpn*I and *Hind*III restriction sites, respectively. The PCR product was digested and cloned into pDrive (Qiagen), creating the plasmid pDM7. The 1010 bp *Pst*I fragment from pUC-Tp [32] containing the trimethoprim (Tp) cassette was inserted in the single *Pst*I restriction site of the *BCAM0223* fragment cloned in pDM7, in the same orientation as the *BCAM0223* gene, creating the plasmid pDM8. This plasmid was introduced into *B. cenocepacia* K56-2 by electroporation. Transformants were selected on LB agar supplemented with 150 mg/L trimethoprim for 48 h at 37°C. To distinguish between single-and double-crossover mutants, trimethoprim-resistant colonies were screened by replica plating for kanamycin sensitivity. The candidate insertion mutants were further characterized by PCR using primers, 223tF2 and 223tR2 (Table S1), which allowed identification of the *BCAM0223*-deficient *B. cenocepacia* K56-2, amplifying the entire gene.

### Hemagglutination Assay

Cultures of *B. cenocepacia* K56-2 and *BCAM0223::Tp* mutant (initial OD_640_ 0.2) were grown for 17 h at 37°C, 60 rpm, under the above established conditions. Cells were harvested by centrifugation, washed once in phosphate buffered saline (PBS) pH 7.4 and resuspended in PBS to an OD_640nm_ of 12 units. The cells (30 µL volumes) were serially diluted (OD_640_ = 1/2, 1/3, 1/4, 1/5, 1/6) and placed in 96-well microtitre plates. To each dilution an equal volume of sheep red blood cells (3% v/v in PBS) was added. The plates were mixed gently with a pipette tip and incubated for 1 h at room temperature. PBS solution was used as a negative control. Each assay consisted of three to five replicates. Hemagglutination was detected by visual inspection of the suspension with direct comparison to the negative control. Each assay consisted of three to five replicates. Results are median values of 3 independent experiments.

### Adherence and Biofilm Formation Assays

Bacterial adherence to extracellular matrix proteins (laminin, fibronectin, vitronectin, collagen types I and IV), was tested as described before with some modifications [28]. Briefly, 96-well polystyrene microplates were coated with 10 µg/mL collagen type IV (in PBS) and 0.5 of µg/mL vitronectin (in water) and placed at 4°C, overnight. Cultures of *B. cenocepacia* K56-2 and *BCAM0223::Tp* mutant (initial OD_640_ 0.2) were grown for 17 h at 37°C, 60 rpm, in microaerophilic conditions, as previous described. Equal amounts (200 µL) were added to the ECM-coated wells. After incubation for 2 h at room temperature, non-bound bacteria were removed and adherent bacteria were fixed with 4% (w/v) paraformaldehyde for 20 min and stained with 1% (w/v) crystal violet for 10 min. After washing twice with PBS, the bound crystal violet was dissolved in 95% ethanol and the absorbance was measured at 595 nm in a microplate reader (Versamax, Molecular devices). Results are median values of at least 5 repeats from 3 independent experiments.

Biofilm formation assays were based on the methodology described by Toole and Kolter [33]. Bacterial cultures were grown under the conditions described above, diluted in LB to an OD_640nm_ of 0.05 and 200 µL of these cell suspensions were inoculated into wells of a 96-well polystyrene microtiter plate. Plates were statically incubated at 37°C for 24 or 48 hours. For biofilm quantification, the culture medium and unattached bacterial cells was removed and wells were rinsed three times with deionized water. Adherent bacteria were stained with 200 µl of a 1% (wt/v) crystal violet solution for 15 minutes at room temperature. After three gentle rinses with deionized water the dye was solubilised with 95% ethanol and measurement of the solution absorbance at 590 nm using a microplate reader. Results are median values of at least 5 repeats from 3 independent experiments.

### Serum Resistance Tests

The resistance of *B. cenocepacia* K56-2 to Normal Human Serum (NHS) was assessed by use of a liquid-phase assay based on a previously described methodology [34]. Briefly, bacterial cultures were grown in microaerophilic conditions previously described. After 17 h of growth, cells were harvested and diluted in PBS to a final concentration of 106 CFU/mL. Then, 10 µL (104 CFU) of cell suspension was added to 30 µL of commercial NHS (Sigma-Aldrich) and 60 µL of PBS, resulting in a final serum concentration of 30%. The mixture was incubated at 37°C for 1 h and then placed on ice. Subsequently, 10 µL of each sample was serially diluted and plated to determine the colony-forming units (CFU). The initial bacterial loading was achieved replacing the NHS for PBS, and 10 µL of the bacterial suspension was serially diluted and plated. As control, we performed all experiments in parallel using heat-inactivated NHS (30 min at 56°C). The percentage of survival was determined as the quantity of bacteria that survived relatively to the initial bacterial loading.

To block the alternative complement activation pathway, factor B-depleted serum (B-NHS) (Quidel) was used. To obstruct both the classical and lectin pathways, NHS was equilibrated in PBS containing 10 mM MgCl_2_ and 10 mM EGTA (LC-NHS) for 15 min on ice before adding the bacteria. Finally to selectively block the classical pathway a C1-q depleted serum (C-NHS) was used.

### Adhesion to and Invasion of Human Bronchial Epithelial Cells

Adhesion and invasion experiments were carried out on non-CF 16HBE14o- and CF CFBE41o- bronchial epithelial cells using an adaptation of the method described by Martin and Mohr [35]. Firstly, cells were seeded on 24-well plates (Orange scientific) (5×105 cells/well) in MEM in MEM medium supplemented and cultured for 24 h at 37°C, in a humidified atmosphere with 5% CO_2_. Then, the cell monolayers were washed with PBS and maintained in MEM medium without supplements. Parental and *BCAM0223::Tp* strains were used to infect host cells at a multiplicity of infection (MOI) of 50∶1 (bacteria per epithelial cell). For adhesion assays, the infected monolayers were incubated at 37°C in 5% CO_2_ for 30 min to allow bacterial adherence. After this, cells were washed three times with PBS and lysed with 200 µL of the buffer (10 mM EDTA, 0.25% Triton X-100) for 30 min at room temperature. The adhered bacteria were quantified by plating serial dilutions of the cell lysates. Results are expressed as a ratio of the wild type and corrected with the initial bacterial dose applied.

For invasion assays, the infected monolayers were incubated at 37°C in 5% CO_2_ for 2 h to allow bacterial entry. After incubation, the monolayers were washed three times with PBS and a combination of amikacin and ceftazidime (2 mg/ml, each) was added and incubated for two hours. The supernatants were then plated to confirm the effectiveness of antibiotic treatment. Finally, the cell monolayers were washed three times with PBS and intracellular bacteria were released using the same lysis buffer used in adhesion experiments. Bacteria were quantified by plating serial dilutions of the cell lysates. The results are expressed as a ratio of the wild type and corrected with the initial bacterial dose applied.

### Confocal Microscopy Examination

*B. cenocepacia* K56-2 and *BCAM0223::Tp* mutant cells were electroporated with pIN29, a plasmid that encodes the red fluorescent protein DsRed [36]. Bacterial adhesion assays were performed as described above except coverslips were introduced in the 24-well plates before the cells seeding. Cells were fixed with 3.7% paraformaldehyde for 20 min before being quenched with 50 mM NH_4_Cl (Sigma) for 10 min and immersed in 0.2% triton X-100 (Sigma) for 5 min. Thereafter, the coverslips were saturated with 0.5% bovine serum albumin (BSA) for 30 min. The immunostaining was achieved by incubation with the primary mouse E-cadherin antibody (clone HECD-1, Takara Bio Inc. Shiga, Japan) (1∶100 dilution) followed by the secondary polyclonal goat anti-rabbit serum coupled to Alexa Fluor 488 (Invitrogen) (1∶500 dilution). Lastly, the coverslips were mounted in Vectashield with DAPI (Vector Laboratories) and samples were examined on a Leica TCS SP5 (Leica Mycrosystems CMS GmbH, Mannheim, Germany) inverted microscope (DMI6000) with a 63× water (1.2 numerical apertures) apochromatic objective [37]. Images of Alexa 488 were captured using 458 nm line from an Ar+ laser and DsRed excitation was performed with the 514 nm line from an Ar+ laser. DAPI excitation was performed using a multiphoton Ti:Sapphire laser (Spectra-Physics Mai Tai BB, 710–990 nm, 100 femtoseconds, 80 MHz).

### Galleria Mellonella Killing Assays

*Galleria mellonella* killing assays were based on the method described by Seed & Dennis [38]. Cultures of *B. cenocepacia* K56-2 and *BCAM0223::Tp* mutant were grown in microaerophilic conditions previously described. Cells were then harvested and ressuspended in 10 mM MgSO_4_ plus 1.2 mg/mL ampicilin. A micrometer was adapted to control the injection volume of a micro syringe. This apparatus was used to inject 3.5 µL of bacterial suspension containing 10 CFU/larvae into the haemocoel of the caterpillars via the hindmost left proleg. Control larvae were injected with the solution 10 mM MgSO_4_ plus 1.2 mg/mL ampicilin. Following injection, larvae were placed in glass Petri dishes and stored in the dark at 37°C. The survival and appearance was recorded at 24 h intervals until 96 h. Larvae were considered dead when they displayed no movement in response to shaking of the petri dish or touch with a pipette tip.

### Statistical Analysis

All experiments were performed a minimum of three times. Relative comparisons were done between corrected values with ANOVA test for significance. A *P* value <0.05 was considered statistically significant.

## Results

### Bioinformatic Analysis of BCAM0223, a New Member of the Trimeric Autotransporter Family

The 4653-bp *BCAM0223* gene from *B. cenocepacia* K56-2 encodes a protein with 1550 residues that contains several domains with homology to members of the trimeric autotransporter adhesin family of surface-attached oligomeric proteins, in particular to the C-terminal translocator domain of YadA, a *Yersinia enterocolitica* trimeric autotransporter adhesin [26]. We have used the web-based daTAA program (http://toolkit.tuebingen.mpg.de/dataa) to confirm that BCAM0223 has, like the other TAA proteins, a head-stalk-anchor modular organization [39]. Using the daTAA program, we also predict the presence of a signal peptide with a cleavage site between amino acids 42 and 43. A BLASTP sequence similarity searches indicated that BCAM0223 shares significant overall amino acid sequence identity with at least three other members of the TAA family. The highest identities (46%) were observed with a protein from *B. cenocepacia* MC0-3 (YP_001777862) and the autotransporter adhesin from *B. cenocepacia* PC184 (ZP_04942611.1) followed by the hemaggluttinin like protein from *B. xenovorans* (Bxe_B2271) (34%). A closer examination of the amino acid identity of BCAM0223 with its counterparts revealed that the higher degree of identity is restricted to the C-terminal domain regions (YadA-head-like domains).

### Transcriptional Organization of the Genes Linked to *BCAM0223*

**Figure 1:**
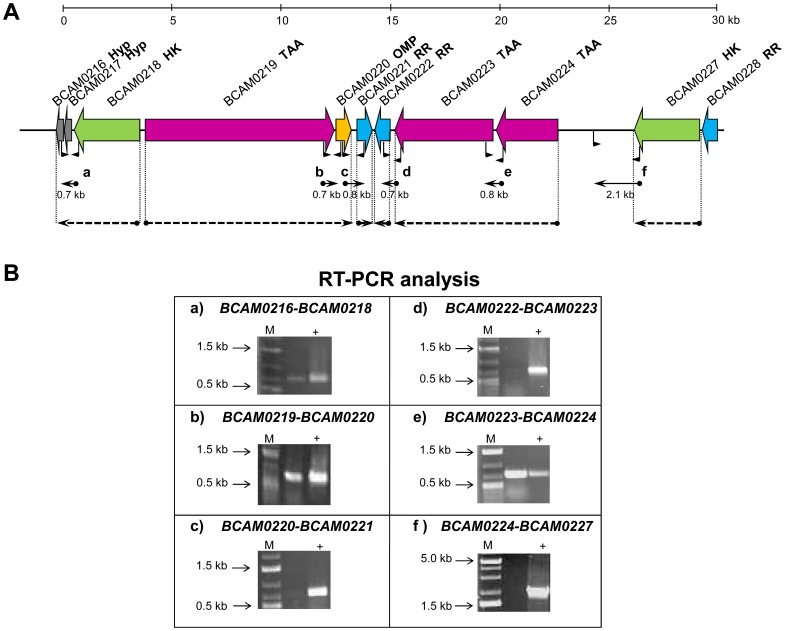
Gene organization of the TAA gene cluster in *B. cenocepacia* K56-2. A ) Genomic organization and operon mapping of the TAA gene cluster. The regions amplified by RT-PCR are indicated by filled arrows and the respective primers location is shown in the genes. The regions co-transcribed are identified with dashed arrows. Hyp – hypothetical protein, HK- histidine kinase, TAA – trimeric autotransporter adhesin, OMP – outer membrane protein, RR – response regulator. **B**) Results from the RT-PCR analysis, showing that no amplification was obtained for regions c), d) and f). Regions a), b) and e) showed amplification indicating that genes are co-trasncribed. Genomic DNA was used as positive control (+). A negative control was performed with RNA without reverse transcriptase, and the result was always negative. M, molecular mass marker.

As previous described, the TAA-encoding gene *BCAM0223* belongs to a cluster of nine genes that we called TAA cluster [28]. To determine the transcriptional organization of this gene cluster, reverse transcription PCR (RT-PCR) experiments were performed in *B. cenocepacia* K56-2. As shown in Figure 1_A, we designed primer pairs that include intergenic regions between the designated open reading frames, allowing the detection of co-transcripts. PCR products of 800 and 700 bp, comprising respectively the intergenic regions of *BCAM0224-BCAM0223*, *BCAM019–BCAM0220* and *BCAM0217–BCAM0218* were detected (Fig. 1_B). In contrast, no PCR products were obtained for the primer pairs designed to amplify the intergenic regions between *BCAM0223*-*BCAM0222, BCAM0227*–*BCAM0224* and *BCAM0220*–*BCAM0221* (Fig. 1_B). Our results thus indicate that the three TAA-encoding genes are organized as two subclusters, consisting of two divergently oriented bicistronic operons, *BCAM0224-BCAM0223* and *BCAM019–BCAM0220*, flanked by regions harboring predicted promoter and terminator sequences. The neighbor genes *BCAM0221, BCAM0222* and *BCAM0227* are organized in monocistronic units (Fig. 1).

### Effect of the *BCAM0223* Mutation on Hemagglutination Ability of *B. cenocepacia* K56-2

**Figure 2:**
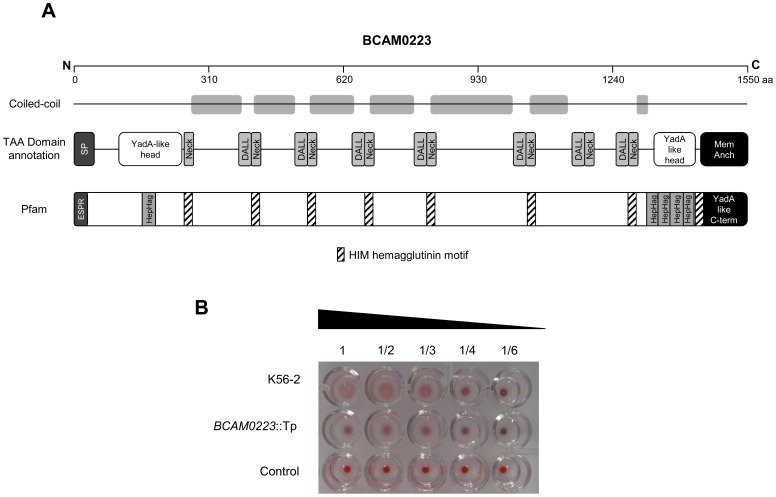
Hemagglutinin motifs in the BCAM0223 protein and effect of the *BCAM0223* mutation on hemagglutination ability. **A**) Schematic illustration of the domain organization and coiled-coil prediction of BCAM0223 protein from *B. cenocepacia* J2315 using data program (http://toolkit.tuebingen.mpg.de/dataa). The protein comprises the typical membrane-anchor domain of TAAs at the C-terminal, a signal peptide (SP) at the N-terminal, two YadA-like heads domains and several connector domains (Neck and DALL). Using Pfam database (http://pfam.sanger.ac.uk/) numerous Hep-Hag repeats and hemagglutinin domains (Him) were found in BCAM0223 protein. Pfam also predicted the YadA-like C-terminal corresponding to the membrane-anchor and an extended SP. **B**) Hemagglutination ability of the wild type *B. cenocepacia* K56-2 and *BCAM0223::Tp* mutant, using 3% sheep red blood cells. PBS was used as a negative control. The highest dilution giving hemagglutination was taken as the titre.

*In silico* analysis of the BCAM0223 amino acid sequence using Pfam protein database [40] revealed significant hits for the presence of eight noncontiguous coiled-coil clusters of HIM motif repeats (Pfam domain PF05662) and five clusters of Hep-Hag motif repeats (Pfam domain PF05658), which are located, respectively, on the neck and head2 domains (Fig. 2_A). HIM and Hep-Hag are short repeat motifs found in bacterial hemagglutinins and invasins [41].

To determine whether the *BCAM0223* gene is related with the hemagglutination ability of *B. cenocepacia* K56-2, we constructed a knockout *BCAM0223* mutant and examined its hemagglutinating activity. The insertional mutation does not affect the growth rate of the mutant relative to the wild-type strain (data not shown). We found that *B. cenocepacia* K56-2 caused hemagglutination of sheep red blood cells after 1 h incubation at room temperature, whereas the *B. cenocepacia* K56-2 (*BCAM0223::Tp*) showed a greater decrease of hemagglutination activity (Fig. 2_B).

### Effect of the *BCAM0223* Mutation on Adherence to Extracellular Matrix Proteins and Biofilm Formation Ability of *B. cenocepacia* K56-2

**Figure 3:**
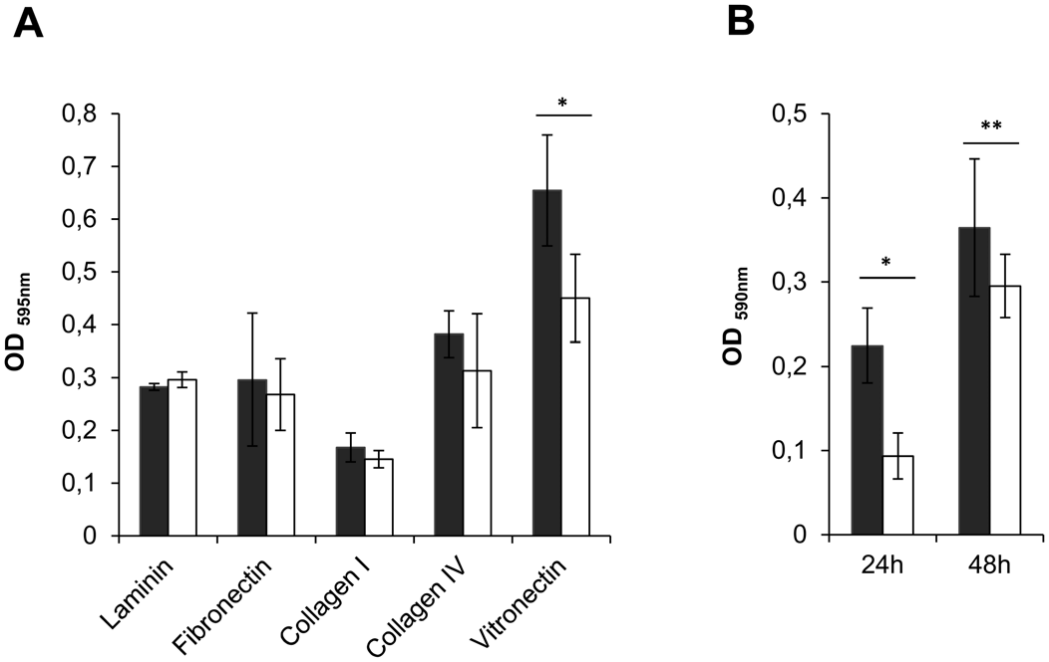
Effect of the *BCAM0223* mutation on adherence to extracellular matrix proteins and biofilm formation. A ) Adherence of wild-type *B. cenocepacia* K56-2 (filled bars) and *BCAM0223::Tp* mutant (open bars) to ECM proteins, laminin, fibronectin, collagen type I and IV and vitronectin. Only the binding capacity to vitronectin was significantly decreased in the mutant (*P<0.001). **B**) Static biofilm formation in polystyrene microtiter plates by wild-type *B. cenocepacia* K56-2 (filled bars) and *BCAM0223::Tp* mutant (open bars) at 24 and 48 h. Biofilm growth was quantified by the solubilization of crystal violet-stained cells with ethanol. Either after 24 h or 48 h incubation, the *BCAM0223* mutant strain exhibited a statistically significant reduction in biofilm formation (*P<0.001 and **P<0.05). All the results are from three independent experiments; bars indicate SD.

A typical feature of TAAs is their ability to adhere to extracellular matrix (ECM) proteins and to promote biofilm formation [12]. Therefore, we examined the ability of the mutant *B. cenocepacia* K56-2 (*BCAM0223::Tp*) to bind immobilized human ECM components, such as laminin, fibronectin, collagen type I and IV and vitronectin. As shown in Fig. 3_A, compared with the wild-type *B. cenocepacia* K56-2, the *BCAM0223*-negative mutant adhered significantly less (*P*<0.001) to vitronectin. In contrast, the wild-type and the mutant did not display significant differences in adherence either to fibronectin, laminin or collagen I and IV (Fig. 3_A).

We next determined the capacity of both wild-type *B. cenocepacia* K56-2 and *B. cenocepacia* K56-2 (*BCAM0223::Tp*) mutant to form biofilms *in vitro* on polystyrene surfaces after 24 and 48 h of incubation in LB medium at 37°C. As shown in Fig. 3_B, either after 24 h or 48 h incubation, the *BCAM0223* mutant strain exhibited a statistically significant reduction in biofilm formation.

### Effect of the *BCAM0223* Mutation on Serum Resistance of *B. cenocepacia K56-2*

**Figure 4:**
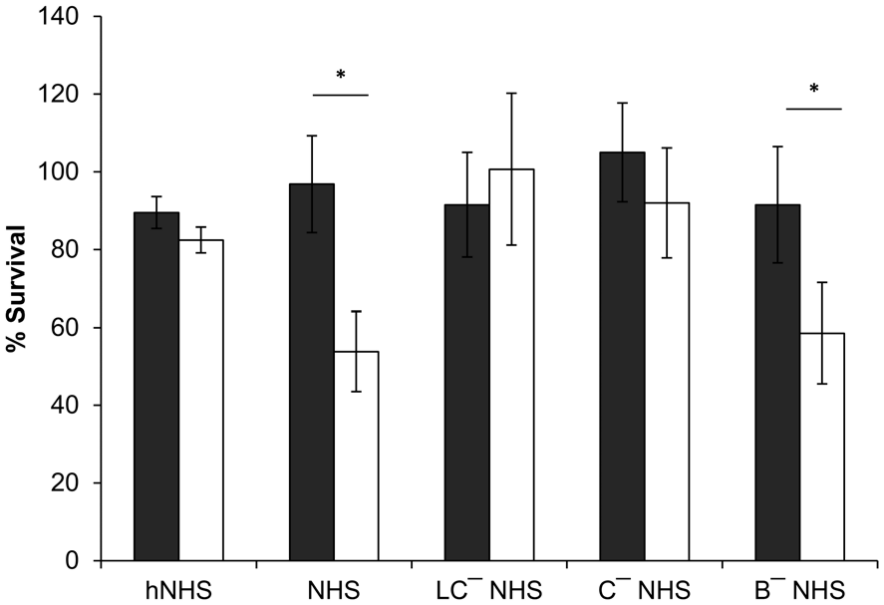
Effect of the *BCAM0223* mutation on serum resistance. The serum bactericidal assay performed using 30% normal human serum (NHS) incubated with *B. cenocepacia* K56-2 (black bars) and *BCAM0223* mutant (open bars) showed significant sensitivity of the mutant to serum killing (*P<0.001). Heat inactivated NHS (hNHS) was used as control of the experiment showing no killing activity. Determination of the pathway involved in serum-killing of *BCAM0223::Tp* mutant, was performed using three distinct sera: NHS containing 10 mM MgCl_2_ plus 10 mM EGTA that block both, the lectin and classical pathways (LC-NHS), a C1q-depleted serum (C-NHS) (blocks classical pathway) (C**-**NHS) and factor B-depleted human serum (B**-**NHS) which contains solely the alternative complement pathway blocked. The wild-type *B. cenocepacia* K56-2 (filled bars) showed resistance to all three sera, whereas *BCAM0223::Tp* mutant (open bars) was resistant to LC-NHS and C**-**NHS but sensitive to B**-**NHS (*P<0.001), suggesting that the killing of the serum-sensitive *BCAM0223::Tp* mutant is mediated by the classical complement pathway. All the results are from three independent experiments.

Bacterial TAAs are often associated with resistance to complement-mediated serum killing, which represent the first line of innate defense against pathogens [42], [43], [44]. This, together with the preceding results showing that the *BCAM0223* mutant has reduced ability to bind vitronectin (Fig. 3_A), a regulator of the complement system [45], prompt us to investigate the functional consequences of the *BCAM0223* mutation on serum resistance. Serum sensitivity assays of *B. cenocepacia* K56-2 and *BCAM0223* mutant were performed using 30% commercial Normal Human Serum (NHS). As shown in Fig. 4, after 1 h of incubation at 37°C, the wild-type strain *B. cenocepacia* K56-2 is highly resistant to killing by NHS (97% survival; *P*<0.001), whereas the *BCAM0223* mutant strain was killed to a significant extent (54% survival). Very little killing was observed in the control assays prepared with heat-inactivated human serum (hNHS) (Fig. 4).

The activation of the complement system can occur via three distinct routes, namely the classical, the mannose binding lectin (MBL) pathway and the alternative pathway. Despite the different activation, classical and lectin pathways have an equivalent progression [46]. To further clarify the role of BCAM0223 in serum resistance, we have conducted additional bactericidal experiments using three different sera, namely, normal human serum containing MgCl_2_ and EGTA to block both the lectin and classical pathways (here abbreviated to LC-NHS), a C1q-depleted serum (C-NHS) (selectively blocks classical pathway) and factor B-depleted serum (B-NHS) that selectively block the alternative pathway. As shown in Fig. 4, when the wild-type and the *BCAM0223* mutant were incubated with LC- NHS little or no killing was observed for both strains. In contrast, the use of a factor B-depleted serum, that selectively blocks the alternative pathway, restored its killing activity against the BCAM0223 mutant (59% survival; P<0.001) but had no effect on the wild-type strain. In addition, to distinguish between the classical and lectin pathways, we conducted assays using the C-NHS serum. No or minimal killing was observed for both strains (Fig. 4). Taken together, these data suggest that the killing of the serum-sensitive *BCAM0223* mutant is mediated through the classical complement pathway but not the mannose binding lectin or alternative pathway.

### Effect of the *BCAM0223* Mutation on Adherence to and Invasion of Cultured Bronchial Epithelial Cells by *B. cenocepacia* K56-2

**Figure 5:**
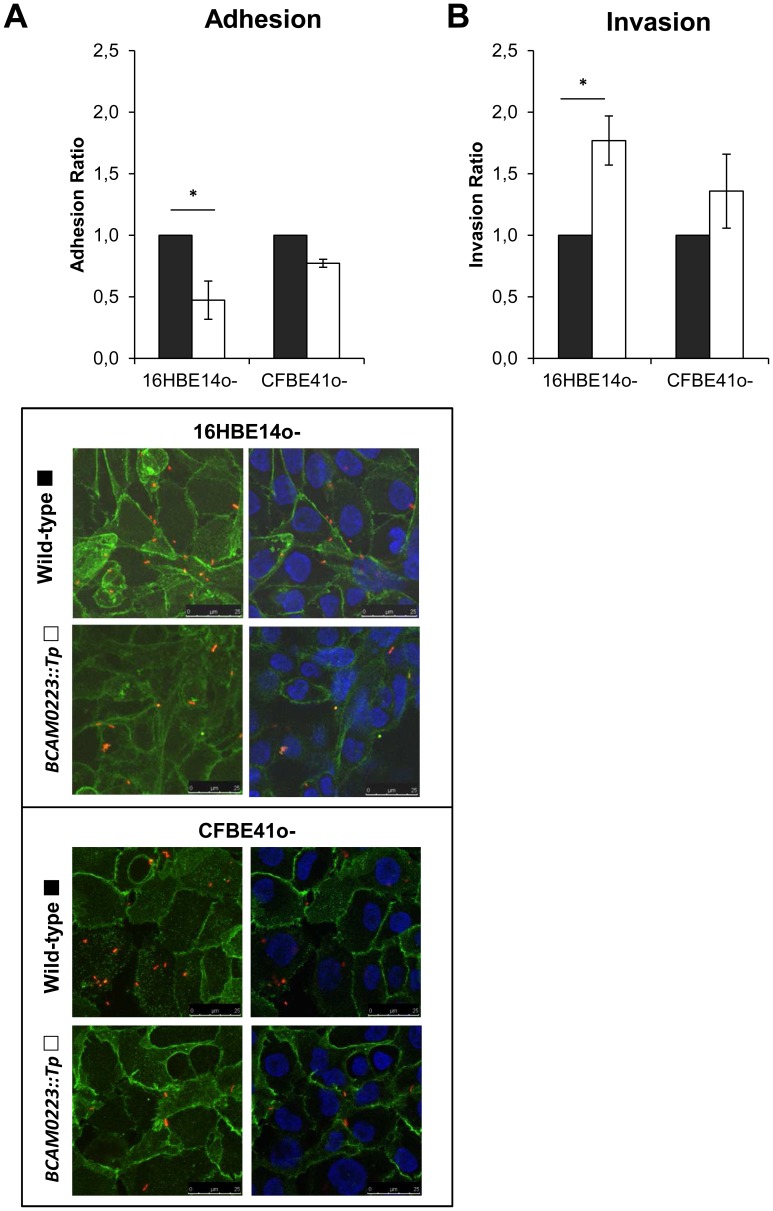
Effect of the *BCAM0223* mutation on adherence to and invasion of **cultured bronchial epithelial cells. A**) Adherence to 16HBE14o- (non-CF) and CFBE41o- (CF) epithelial cell lines by *BCAM0223::Tp* mutant (open bars) expressed as ratio of *B. cenocepacia* K56-2 (black bars) adherence. *BCAM0223*-negative mutant adhered less efficiently than the wild-type strain, particularly to the non-CF cell line (*P<0.01). Fluorescence confocal microscopy images of *B. cenocepacia* K56-2 and *BCAM0223::Tp* mutant adhered to 16HBE14o- (non-CF) and CFBE41o- (CF) epithelial cell lines. Left and right panels correspond to representative images obtained from 0.4 µm confocal slices of cells monolayers. The green color reflects the eukaryotic cells plasma membranes labeled with Alexa Fluor® 488 anti-mouse E-cadherin (green) while the blue color represents the nucleus stained with DAPI. The red color represents the bacteria labeled with DsRed. **B**) Invasion of 16HBE14o- (non-CF) and CFBE41o- (CF) epithelial cell lines by *BCAM0223::Tp* mutant (open bars) expressed as ratio of *B. cenocepacia* K56-2 (black bars) internalized. The *BCAM0223*-negative mutant displayed an enhanced invasive capacity on both cell lines, particularly to the non-CF cell line (*P<0.001), when compared with the wild-type strain *B. cenocepacia* K56-2. All the results are from three independent experiments.

As their name suggest, TAAs such as YadA, HadA, NadA, YadBC and ApiA have been directly associated with bacterial adherence to and invasion of mammalian host cells [14], [18], [47], [48], [49]. Therefore, we have used a CFU-based quantification assay to evaluate the *in vitro* capacity of the mutant *B. cenocepacia* K56-2 (*BCAM0223::Tp*) to adhere to monolayers of two human bronchial epithelial cell lines (16HBE14o- and CFBE41o-) which have respectively a non-Cystic Fibrosis (CF) and CF phenotype [30], [31]. As shown in Fig. 5_A, the *BCAM0223*-negative mutant adhered less efficiently than the wild-type strain, particularly to the non-CF 16HBE14o- cell line (approximately 50% reduction; *P*<0.01). Analysis of the stained cells by confocal immunofluorescence microscopy confirm this result, revealing that, compared with the wild type, the *B. cenocepacia* K56-2 (*BCAM0223::Tp*) mutant is impaired in its adherence to bronchial epithelial cells (Fig. 5_A, bottom panel). Thus, these *in vitro* results suggest that the TAA BCAM0223 is needed to maximize adhesion to human bronchial epithelial cells.

Furthermore, we have also evaluated by an antibiotic protection assay, the ability of the *B. cenocepacia* K56-2 (*BCAM0223::Tp*) mutant to invade host epithelial cells. Surprisingly, it was observed that in contrast to the adherence, the *BCAM0223*-negative mutant, compared with the wild-type strain *B. cenocepacia* K56-2, displayed an enhanced invasive capacity on both cell lines, particularly to the non-CF 16HBE14o- cell line (approximately 2 fold increase; *P*<0.001) (Fig. 5_B). Taken together, our results suggest that the TAA BCAM0223 is required for *B. cenocepacia* maximal adherence to but not for invasion of cultured human respiratory epithelial cells.

### The Mutant *B. cenocepacia* K56-2 (*BCAM0223::Tp*) Exhibits Reduced Virulence in a *Galleria Mellonella* Model of Infection

**Figure 6:**
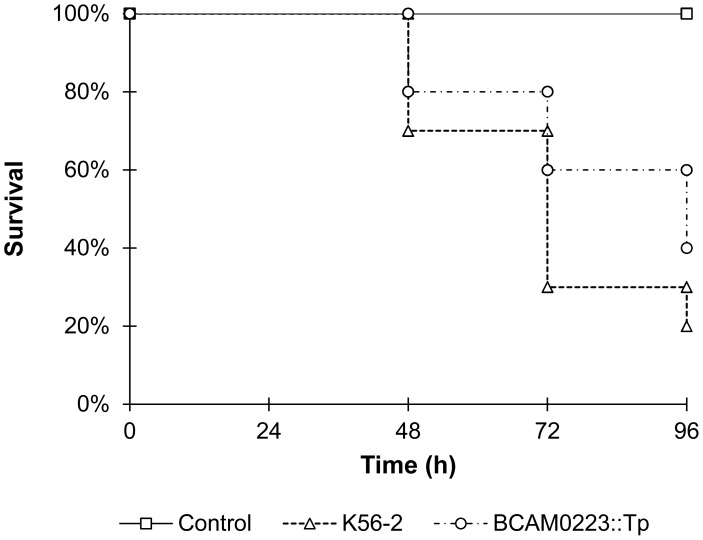
Kaplan-Meier graph of *Galleria mellonella* survival after injection (10 CFU/larvae) with wild-type *B. cenocepacia* K56-2 and the *BCAM0223::Tp* mutant (*P*<0.01, for comparison of the wild-type and the mutant). Uninfected larvae injected with NaCl 0.9% were used as control. Results represent means of three independent determinations for 10 animals per treatment.

Finally, in order to evaluate the effect of the *BCAM0223* mutation on *B. cenocepacia* virulence, we compared the ability of the wild-type and the *BCAM0223::Tp* mutant to kill the larvae of the insect model *Galleria mellonella*. As shown in Fig. 6, 96 h following infection, the *BCAM0223::Tp* exhibited attenuated (20%) killing ability in comparison to the wild-type *B. cenocepacia* K56-2 (*P*<0.01), indicating a role of BCAM0223 in virulence.

## Discussion

Our work has focused on one category of virulence determinants, the trimeric autotransporter adhesins (TAAs). To date, very little is known about their mode of action and their contribution for the overall virulence of Bcc members. In a previous study, we have identified *in silico* seven TAA-encoding genes in the genome of the epidemic strain *B. cenocepacia* J2315 [13], [28]. Among those, three TAA-encoding genes (*BCAM0219*, *BCAM0223* and *BCAM0224*) are located within a cluster (named TAA cluster) on chromosome 2 of *B. cenocepacia* J2315. The TAA cluster span approximately 30 kb and includes, beyond the three TAA-encoding genes, six other genes respectively coding for one lipoprotein, two sensor histidine kinases, and three response-regulators [28]. Moreover, it has been suggested that this cluster might represent a prototype of a two-component signal transduction cascade, where the two histidine kinases enable bacteria to sense specific environmental stimulus thereby transmitting the signal to response regulators that ultimately control the expression of the TAA-encoding virulence genes [28]. This observation is consistent with recent findings that characterize the sensor kinase BCAM0227 as a novel sensor of the quorum-sensing signal molecule *cis*-2-dodecenoic acid (BDSF) [50].

In this work, we have continued the characterization of the TAA cluster. Firstly, we have used RT-PCR to determine the transcriptional organization of the cluster. The nine clustered genes were shown to be organized in six transcriptional units. The four virulence effector genes of the cluster were grouped in two divergent sub-clusters, grouping respectively, two TAAs (*BCAM0224* and *BCAM0223*) and one TAA together with a lipoprotein (*BCAM0219* and *BCAM0220*) (Fig. 1). We hypothesized that these putative virulence genes are expressed under certain external environmental conditions sensed by the two histidine kinases. Interestingly, in a comparative transcriptomic analysis of two clonal variants of *B. cenocepacia* (IST439 and IST4113), *BCAM0219* and *BCAM0223* are proposed to be up-regulated in IST4113, the clinical isolate associated with a multiple resistance phenotype and long-term persistence [51]. In addition, a study by Bernier and Sokol [52] reveals that the overexpression of *BCAM0223* enhances survival of *B. cenocepacia* in the lungs of a CF rat model.

Taken together, these findings prompted us to undertake this study, in which we functionally analyzed and compared wild-type with mutated *B. cenocepacia* K56-2 (*BCAM0223::Tp*), aiming to investigate the role of this annotated TAA-encoding gene in the overall virulence of *B. cenocepacia* J2315. Unfortunately, despite many efforts, we failed to clone the entire *BCAM0223* gene and hence we were unable to perform the mutant complementation analysis. However, as shown in figure 1, *BCAM0223* is the downstream gene in a bicistronic operon with *BCAM0224*, so its disruption should not have a polar effect on the expression of the downstream gene, *BCAM0222*. RT-PCR assays using mRNA isolated from the *B. cenocepacia* K56-2 (*BCAM0223::Tp*) mutant confirm the lack of polarity (data not shown).

Hemagglutination is one of the various functions attributed to TAAs [53], [54]. Here we demonstrate that the *BCAM0223* mutation caused a marked decrease in the hemagglutination-positive phenotype of *B. cenocepacia* K56-2. This finding is consistent with the *in silico* analysis of BCAM0223 in which various hemagglutinin motifs (8) were predicted (Fig. 2). It will be interesting to see whether these motifs have immunogenic properties making them useful candidates to develop a protective vaccine against *B. cenocepacia*.

Next we analyzed the impact of the *BCAM0223* mutation on adherence to immobilized extracellular matrix proteins (ECM). Among the five ECM proteins tested, the *BCAM0223::Tp* mutant showed only a significantly reduced adhesion to vitronectin (Fig. 3_A). Further work is needed to prove direct association between vitronectin and BCAM0223 and ultimately map the BCAM0223 region(s) involved in such interaction(s). Notably, vitronectin has been described to participate in the adhesion of Gram-negative and Gram-positive pathogens to host cells and exists in high amounts in an insoluble form in lungs tissues [55]. Moreover, vitronectin has two separate binding sites for pathogens and host epithelial cells, thereby serving as a bridge to connect host and bacterial cells [56]–[58]. It has been demonstrated that other TAAs are also able to bind vitronectin, such as DsrA from *Haemophilus ducreyi* [59], Hsf from *H. influenzae* type b [60] and UspA2 from *Moraxella catarrhalis* [61].

Besides the reduced capacity to adhere to vitronectin, the *B. cenocepacia* K56-2 (*BCAM0223::Tp*) mutant is also impaired in biofilm formation on polystyrene surfaces (Fig. 3_B). Our study thus suggests that, like many other TAAs [20], [23], [41], BCAM0223 may be necessary for biofilm formation. In general, the production of biofilm may contribute for long-term persistence of virulent pathogens in their host [62]. This is probably the case of Bcc long-term infection, where the establishment of biofilms in the respiratory tract of in CF patients may confer antibiotic resistance and protect bacteria from attack by the host’s immune system [6].

Several TAAs have been described to directly bind various factors of the complement system, thereby representing critical components associated with bacterial complement escape [46]. These include, among others, UspA1 and UspA2 from *Moraxella catarrhalis* that have been reported to bind C4b-binding protein [63], YadA from *Y. enterocolitica* reported to bind factor H (FH), the negative regulator of the alternative complement pathway [64] and EibD from *E. coli*, which binds IgG and IgA through its coiled-coil stalk domain [65]. However, unlike other bacterial pathogens, in Bcc bacteria, the mechanisms of human serum resistance are poorly understood. To date, only the O-antigen portion of LPS has been described to confer serum-resistant to *B. cenocepacia* K56-2 [66]. In this study, we investigated the impact of the *BCAM0223* mutation on serum resistance. Our results show a marked reduction (50%; *P*<0.001) in the serum resistance of *B. cenocepacia* K56-2 (*BCAM0223::Tp*) mutant. Furthermore, we also present evidences that the killing of BCAM0223 mutant required the classical complement pathway but not the lectin or alternative pathways (Fig. 4). Interestingly, as stated above, it has been shown that the disruption of *BCAM0223* gene caused a marked inhibition of bacterial adhesion to vitronectin. Since vitronectin, beside its function as a cell-adhesion molecule, is also a human plasma protein, acting as potent inhibitor of complement activation, we hypothesized that BCAM0223 by interacting with vitronectin can confers protection to *B. cenocepacia* K56-2 against the membrane attack complex (MAC) of complement. Further work is needed to elucidate the molecular details of serum resistance mediated by BCAM0223, namely the stage of the classic complement cascade that is blocked. These studies will analyze the rate and nature of complement components deposited on the bacterial cell surface of both wild type and *BCAM0223* mutant. Ultimately, we intend to map the BCAM0223 region(s) involved in such interaction(s).

Bcc strains were able to adhere to and invade cell culture monolayers of human respiratory epithelial cells with various efficiencies. However, many aspects about the molecular mechanisms underlying such events, particularly the nature of bacterial ligands and their cognate host receptors, remains to be elucidated [8]. In this study, we examined the functional consequences of the *BCAM0223* mutation on *in vitro* bacterial adherence to and invasion of two human bronchial epithelial cell lines (16HBE14o- and CFBE41o-) which have respectively a non-Cystic Fibrosis (CF) and CF phenotype [30], [31]. Results obtained revealed a reduction of adherence of the *BCAM0223::Tp* mutant compared to the parent strain. This observation is particularly significant for host cells with a non-CF phenotype (Fig. 5_A), suggesting that, at least *in vitro* and under the experimental conditions used, CF cells may have exposed on cell surface, a lower level of BCAM0223 adherence receptors. On the other hand, surprisingly, we found that while with a reduced adherence phenotype, the *BCAM0223::Tp* mutant invades host cells better than the wild-type strain (Fig. 5_B). The reason for this is still unclear. We hypothesized that since *B. cenocepacia* K56-2 has multiple multifunctional TAAs and other conventional adhesins as cell-surface components, including BCAM0219 and BCAM0224, it is perhaps not surprising that the ablation of one of them, such as BCAM0223, could further improve the function of other specialized invasion factors. Moreover, it also remains possible that the impaired cell adhesion observed in the *BCAM0223*::Tp mutant appeared as an indirect effect due to its increased invasion ability. In this context, further works will be required to clarify this observation and assign the function of the various *B. cenocepacia* TAAs in host cell invasion.

Finally, we have used the host *Galleria mellonella* wax moth larvae as a model for assessing the *in vivo* role of BCAM0223 in *B. cenocepacia* virulence. We have observed that although moderated, the absence of BCAM0223 impairs the virulent phenotype of *B. cenocepacia* K56-2 (Fig. 6). Thus, we believe that BCAM0223 should be included in the large arsenal of virulence factors encoded by *B. cenocepacia*, thereby contributing to its overall pathogenicity.

In conclusion, in this study we have performed the functional analysis of a TAA mutant of *B. cenocepacia* K56-2, which is defective in one (*BCAM0223*) of the seven predicted TAA genes encoded by its genome. The phenotypic analysis of this mutant strongly suggests that BCAM0223 is a multi-faceted adhesin that possesses hemagglutinin activity and represents a vitronectin-binding protein that may directly contributes to the virulence of *B. cenocepacia* K56-2, complement evasion and bacterial binding to epithelial host cells.

## Supporting Information

Table S1 Oligonucleotide primers used in this study.
(PDF)
