# Supplementary material for: A BCAM0223 Mutant of Burkholderia cenocepacia Is Deficient in Hemagglutination, Serum Resistance, Adhesion to Epithelial Cells and Virulence
Source: PLoS One. 2012 Jul 25;7(7):e41747. doi: 10.1371/journal.pone.0041747 (PMC3404963; doi:10.1371/journal.pone.0041747)
Supplement: Table S1 — Oligonucleotide primers used in this study. (PDF) [file pone.0041747.s001.pdf]

Primer	Sequence (5'-3')
223fF1	GGGGTACCATCGCGATAACGCAGTGTCTGT
223fR1	TTTAAGCTTTCAGCTTGTCGTTGAGCTTCGT
223tF2	AACAAGACCTATCGCAGCGTGTG
223tR2	CGCGATCGGGTCAGAACTCAT
216-218fwd	TCGCGATTCATTGTGCGTTTCC
216-218rev	TGAGTTCGAACGCTATCGGCAAAG
219-220fwd	TCAACATCCGCGACGGTATCAAC
219-220rev	CTTGCCGCACATGAACGTGATT
220-221fwd	ATCAACAGCGGCATCGAATTCC
220-221rev	ACTTGCTGACGATGCACTCGAT
222-223fwd	GATGCGAATGGGGAAGGTTCTGTT
222-223rev	AAGAATGCGTATGCCGGTATCG
223-224fwd	TTCGAATGCGACTTCGCCGTTT
223-224rev	ATCGCGATAACGCAGTGTCTGT
224-225fwd	GACGAACCTCTCGAATGCGACTTT
224-225rev	GCAAGCAACTGAACGCGAACAA
225-227fwd	ATTTGCGCGTATGCATGCGT
225-227rev	CACTGCGCCATGAACTCCATT
